# Content validation of the Kamath and Stothard questionnaire for carpal tunnel syndrome diagnosis: a cognitive interviewing study

**DOI:** 10.1186/s12955-020-01614-7

**Published:** 2020-11-07

**Authors:** Armaghan Dabbagh, Joy C. MacDermid, Tara L. Packham, Luciana G. Macedo

**Affiliations:** 1 School of Physical Therapy, Faculty of Health Sciences, Elborn College, Western University, London, ON Canada; 2 McMaster University, Hamilton, ON Canada; 3 Roth McFarlane Hand and Upper Limb Centre, St. Joseph’s Hospital, London, ON Canada

**Keywords:** Carpal tunnel syndrome, Diagnosis, Kamath and Stothard questionnaire, Content validity, Cognitive interviewing

## Abstract

**Background:**

Accurate diagnosis of carpal tunnel syndrome (CTS) is essential for directing appropriate treatment; and for making decisions about work injury claims. The Kamath and Stothard Questionnaire (KSQ) is a self-reported tool used for the diagnosis of CTS. Comprehensibility and comprehensiveness of this questionnaire are critical to diagnostic performance and need to be established. The purpose of the study was to describe how potential respondents, clinicians, and measurement researchers interpret KSQ questions in order to identify and resolve potential sources of misclassification.

**Methods:**

Hand therapists, measurement researchers, participants with CTS, and a control group were interviewed using cognitive interviewing techniques (talk aloud, semi-structured interview probes) in Hamilton, Canada. All interviews were recorded and transcribed verbatim. A directed content analysis was done to analyze the interviews using a previously established framework.

**Findings:**

Eighteen participants were interviewed. Areas, where questions were unclear to some participants, were recorded and categorized into five themes: Clarity and Comprehension (52%), Relativeness (38%), Inadequate Response Definition (4%), Perspective Modifiers (4%), and Reference Point (2%). Respondents also identified several symptoms of CTS that are not covered by the KSQ that might be of diagnostic value, e.g., weakness and dropping items.

**Conclusion:**

The content validity of the current iteration of the KSQ was not established. The problematic questions identified in the study have been reported to have low specificity and negative predictive values in a previous quantitative study. The content validity issues identified may explain the poor performance. Recommendations were made to modify the wording of the KSQ and the potential addition of three new questions. Future studies should determine whether the modified questionnaire can provide better diagnostic accuracy and psychometric properties. The results of this study may assist in ruling in or out CTS diagnosis to a wide variety of target audience, such as hand specialists, physical and occupational therapists, as well as family doctors.

## Background

Carpal tunnel syndrome (CTS) is a condition caused by the entrapment of the median nerve within the carpal canal and constitutes the most common compression neuropathy, forming 90% of all peripheral neuropathies [1, 2]. CTS has a rough prevalence of 4–5% in the adult working population, and the prevalence is even higher in women aged 40–60 years, reaching to 9.2% [3]. CTS ranks amongst the top leading causes of days lost from work and productivity loss, affecting 0.7 per 10,000 workers annually [4]. In the occupations that impose a high physical burden on the upper extremity, such as ‘administrative and secretarial’ and ‘skill trades’ the annual incidence of CTS is as high as 82 and 136 per 10,000 workers, respectively [5]. Signs and symptoms of CTS are predominantly represented by pain, numbness, tingling, and weakness of the wrist and hand area innervated by the median nerve (lateral 3.5 digits) [2, 6]. Accurate diagnosis of CTS is essential for directing appropriate treatment, and decisions about work injury claims. Although several tools exist for the diagnosis of CTS, there is no overall consensus on the best choice of clinical measures that assess the existence and severity of CTS [4]. Recent clinical practice guidelines by the American Academy of Orthopedic Surgeons have suggested that multi-item diagnostic tools should be used in the diagnosis of CTS [4].

The Kamath and Stothard Questionnaire (KSQ) is a brief version of Levine’s questionnaire [7] for the diagnosis of CTS [8] (see “Appendix 1” for the KSQ). Kamath and Stothard stated they had developed this questionnaire based on the questions from the Levine et al. [7] CTS outcome measure; however, the questions and scoring are different, so it is unclear how it was used. The KSQ consists of nine questions with yes/no/not applicable answers that are summed to generate a total score [8]. One study suggested that is has a high sensitivity (85%) and positive predictive value (90%) compared to nerve conduction studies [8] in diagnosing CTS. According to a study conducted by Bridges et al. [9], those scoring higher than six on the KSQ, do not need any further testing for the confirmation of CTS diagnosis (87% specificity). In another study by Sangram et al. [10] good to excellent sensitivity has been reported for the KSQ scores of 5 and above. In a recent systematic review on the diagnostic accuracy of scales, questionnaires, and hand symptom diagrams for CTS diagnosis, it was shown that although the KSQ has promising diagnostic accuracy, it has never been validated with the input from people who live with the actual experience of CTS [11]. We believe that establishing the content validity of this questionnaire might lead to improved diagnostic accuracy.

Content validity is defined as the ability and adequacy of a questionnaire to measure all aspects of the construct that is being tested [12]. Cognitive interviewing (CI) is one of the methods to qualitatively assess the content validity of a questionnaire [12]. CI evaluates how potential respondents interpret and calibrate responses to items on a questionnaire, and can potentially identify sources of error in the questionnaires [13]. This technique mainly focuses on interpreting the thought and decision making processes that are used by the respondents to answer the questions [13]. Identification of errors in questionnaires and consequent item revision would potentially lead to improved psychometric properties of any questionnaire [13].

The aim of this study was to describe how persons experiencing hand symptoms and expert hand therapists or researchers understand and calibrate responses to the KSQ in order to resolve potential sources of misclassification.

## Methods

### Study design and ethics

This is a cross-sectional study, approved by the Hamilton Integrated Research Ethics Board (HIREB) at Hamilton, ON, Canada (project #5543), and all participants provided informed consent before their participation.

### Sampling and recruitment

A purposeful sampling technique was administered to ensure that information-rich participants for the study were included [14, 15]. As a result of this maximum variation technique, included 18 participants consisted of two groups as follow:

(A) *Experts group:* The experts group included (1) Hand therapists who are the experts in this field and have a degree in physiotherapy (PT), occupational therapy (OT), or medicine (i.e., hand surgeon, or orthopedic surgeon); and at least two years of work experience, and (2) Graduate students, enrolled in either a master or Ph.D. of Rehabilitation Science at McMaster University and a professional background of OT or PT, who had knowledge about questionnaire construction and could provide data about common errors such as confusing, misleading, and double-barreled questions. Hand therapy clinicians and graduate students with a known interest in diagnosis and measurement theory, or CTS were approached and asked to participate in this study. In addition, recruitment emails were sent to local hand interest group clinicians by one of the research team members (TP), with an invitation to participate in the study.
(B) *Patients group:* Patients were eligible to participate if they met one of the following criteria: (a) mild, moderate or severe CTS diagnosed by electromyography, nerve conduction studies, or clinical examination tests; or (b) neurologic, musculoskeletal or vascular manifestations of upper extremity symptoms (e.g. cervical radiculopathy, shoulder arthritis, etc.). This data was used to make comparisons with the data gathered from the CTS group, in keeping with the intended use of this tool for diagnosing purposes. For the recruitment of the patients, a multi-faceted strategy was administered as follows. Clinicians with interests in hand rehabilitation were emailed and provided with explanations about the study and a recruitment flyer. They were asked to refer patients (if interested in participation) with target diagnosis/signs and symptoms to the researchers. Posters were put up in several buildings of the McMaster University’s main campus. The recruitment flyers were shared on a few local Facebook groups. Potential participants were asked to contact the research team.

No exclusion was made based on age or gender in any of the groups or based on the educational level of the participants in the patients’ group. Exclusion criteria for the CTS group were persons with potentially confounding co-morbidities: any generalized neuropathy such as diabetes mellitus, renal transplant patients, rheumatoid arthritis, hypothyroidism, or connective tissue diseases. All of the participants were required to speak and read English to be eligible to participate in this study.

### Demographic data

Information regarding participants’ age, gender, duration and severity of CTS symptoms, or professional backgrounds were collected by the interviewer. In order to maintain the anonymity of the participants, all of the documents that included identifying information such as the audio files and the demographic data collection sheets were coded with a pre-determined coding system. The coding of the experts group was EO for OT experts, EP for PT experts, and ES for student experts. The patients group codes in CTS and Non-CTS groups were PC1, PC2… and PNC1, PNC2…, respectively.

### Cognitive interviewing procedure

A single, face-to-face session of cognitive interviewing was conducted with 13 of the participants, and the remaining participants (n = 5) were interviewed over the phone. The CI sessions lasted for 0.5–1 h and were audio-recorded and consequently transcribed for analysis. All of the interviews were conducted by one interviewer (AD), an experienced PT with training in CI methods [12, 16]. Two practice interviews were conducted with potential experts group participants before the beginning of the study, to practice the semi-structured interview guide. These two practice interviews were not included in the analysis.

Two cognitive interviewing elements that were implemented in this study were: (1) Think-aloud, meaning that the participants were encouraged to articulate their thoughts while answering the questions; and (2) Verbal probing, where the interviewer actively tried to collect detailed information by probing the participants’ responses [13]. The verbal probing method was done using both pre-determined open-ended questions (“Appendix 2”), and additional probes specific to the participants’ responses. At the end of the interviewing session, participants were asked to provide general feedback on the questionnaire, using questions such as “Is there anything about your symptoms that you feel was not covered by these questions today?”

### Sample size and saturation

We pre-determined the data saturation point as the point where three consecutive interviews did not result in any new data from the participants. To achieve this goal, we analyzed the transcripts from each participant right after the interview was done, and determined if we needed to do more interviews or not. The data saturation was achieved at 18 participants enrolled.

### Data analysis

Demographic data were entered into STATA, version 14 [17], to generate descriptive statistics of the personal characteristics of the respondents. A directed (also known as deductive) [18] qualitative content analysis technique was used to interpret, analyze, and summarize the data from the interviews [14, 19]. Interviews were transcribed after each CI session and were imported into Microsoft Word in a question-by-question format. Common themes and issues were noted from each question and were categorized in one or several of the following pre-determined codes: Clarity/Comprehension, Relevance, Inadequate Response definition, Reference Point, Perspective Modifiers. This coding system was developed by one of the authors of this study (JM) [16]. No additional codes emerged during the process of transcribing and categorizing the sources of response error. The coding process was done by the first author (AD) and reviewed for accuracy by another one of the research team members (JM).

## Findings

### Participants

Eighteen voluntary participants contributed to this study. The expert group included eight participants with backgrounds in PT and OT, and mean work experience of 11.6 years (SD = 6.4). The patient group consisted of ten participants at different stages of CTS and different types of upper extremity diseases. The mean age of the participants was 35.2 years (SD = 9.9), and the mean duration of CTS symptoms was 3.9 years (SD = 3.3). Table 1 illustrates the detailed demographic information of the participants.

**Table 1:** Participants demographic information

Variable	Mean ± SD	Range	Frequency (n)	Percentage (%)
*Participants with or without CTS (n* = *10)*				
Age (y)	35.2 ± 9.9	19–48		
Duration of symptoms (y)	3.9 ± 3.3	0.5–12		
Severity of CTS			Mild, n = 2	
			Moderate, n = 2	
			Severe, n = 1	
			Participants without CTS, n = 5	
*Type of occupation*				
Manual			5	50
Not manual			4	40
No occupation			1	10
*Gender*				
Women			8	80
Men			2	20
*Experts group (n* = *8)*				
Work experience (y)	11.6 ± 6.4	4–20		
*Profession*				
Occupational therapy			2	25
Physiotherapy			6	75
*Level of education*				
B.Sc.			3	37
M.Sc.			2	25
Ph.D.			3	37

*SD* standard deviation, *y* years, *CTS* carpal tunnel syndrome

### Problematic themes identified by cognitive interviewing

All of the participants expressed some interpretive dissonance when responding to the questions. Overall, the content analysis of the transcribed interviews revealed the participants indicated 80 significant sources (Table 2) of response errors that were categorized into the following themes. Table 2 demonstrates the errors identified by the cognitive interviews, sorted based on the themes and participant groups.

1. *Clarity/comprehension*: This theme was the most common (52%), and raised when a word or the entire question was perceived as being vaguely worded, causing different interpretations of the same question or difficulties in understanding [16]. Clarity issues are described relative to each question below.

The concept of *tingling* appeared to be problematic and misleading to five participants in both groups for question #2: Has tingling and numbness in your hand woken you during the night? An example of this dissonance is a participant who initially answered ‘no' to this question and asked for clarification. When she was explained about the meaning of tingling, she changed her answer to ‘yes.’ Another participant also stated, *“I do not have numbness, no, but tingling… sometimes at night, it burns, I do not know how I use the word "tingling," I cannot define this word"—PNC3.*

**Table 2:** Cross-item analysis summary of the response errors

Questions themes	Q1	Q2	Q3	Q4	Q5	Q6	Q7	Q8	Q9	Total number of errors for each category across all of the questions
C		E:2	E:5	E:5	P:3	E:4			E:6	E:25
		P:3	P:3	P:5		P:1			P:4	P:16
										T:41
R	E:6	E:5	E:3			E:6	E:2	E:1		E:23
	P:1	P:1	P:1			P:5				P:8
										T:31
IR				E:3						E:3
RP						P:1			P:1	P:2
PM	E:1							E:2		E:3
Total number of errors for each question:	E:7	E:7	E:8	E:8		E:10	E:2	E:3	E:6	E:54
	P:1	P:4	P:4	P:5	P:3	P:17			P:5	P:26
										T:80

*E* experts, *P* patients, *T* total, *C* comprehension/clarity, *R* relevance, *IR* inadequate response definitions, *RP* reference point, *PM* perspective modifier

Aside from the concept of *tingling* noted in the previous question, the word *‘pronounced’* in the third question seemed to be difficult to interpret, with nearly half of all participants (n = 8) identifying concerns, as illustrated by the following examples. *“I think the word ‘pronounced’ is not necessarily an easy word to understand. I think people would understand the word ‘worse or more noticeable’ easier than pronounced”—ES3;* and*: “What do you mean by the word pronounced here? I am not sure if I understood this question completely”—PNC2.*

Question #4 had the highest occurrence of misinterpretations and difficulties in comprehension (n = 10). The main problem was the phrase *‘trick movements,’* and most of the participants (mainly in the clinicians’ group) recommended to reword this phrase to *‘change in posture’.* As an example: *“I do not know what the word trick movement means. I think this is a difficult question to answer. We use tricky movements to show off something like a magic trick, not like a regular movement”—* EO1.

In addition to the phrase ‘trick movements’, two of the participants in the CTS group could not comprehend the meaning of *“going away from your hands”: “I have no idea what this is asking. Do I have any trick movements to make it go away or to start tingling? This is what I do not understand. Also, I do not believe it is a trick movement; it is just a movement"—*PC5.

Although seemingly a straightforward question, three of the participants made a mistake about the anatomical location of their little fingers in the fifth question: *“my little finger is the third one, the one in the middle”—*PC4; and *“I think my little finger could be any of the fingers, other than my thumb. It was not easy for me to answer this question and I think there might be different opinions about little finger”—*PNC3.

Five participants, including four experts and one patient, raised issues of Clarity regarding the sixth question. One of the experts stated, *“The word ‘presented’ I do not think that is the right word to use… does it mean that it just happened, or did you feel it?"—*EP3 A participant in the non-CTS group also identified a lack of clarity around the included activities. *“I am not sure if I understood ‘knitting’ but steering the car and reading the newspaper part were easy to understand for me. Also, when I use my laptop, it makes me painful”—*PNC2.

Two Comprehension issues raised by 10 participants on question #9 were the words ‘*helped’* and *‘splint,’* as these did not appear to have a common interpretation*.* The participants in the expert group believed that rephrasing the question would make it more understandable and proposed alternate wording.

“I always ask that question, and I like it. However, you’ve got to define what is a splint; people ask me this all the time. Is it a brace, is it a glove? I think splint is usually considered to be custom-made and made at a hospital; versus a brace which you can pick up from a shelf. I think it is a valuable question by just modifying it to: have your symptoms improved with using support on your wrist?"—EP3

2. *Relevance*: 38% of concerns raised were coded as this theme, defined as when people had difficulties relating the questions to their condition or individual lives [16]. The concepts of ‘*day’* and ‘*night’* in the first, second and third questions were mentioned 17 times by the participants across all of the groups (question #1: *has pain in the wrist woken you at night*). The participants mentioned that asking about specific times (day and night) would cause misinterpretation of the questions since not everybody sleeps at night (i.e., night shift workers, nurses, doctors); therefore, it narrows down the relevancy and applicability of the questionnaire.

"Using the term night in this question could be problematic… I was thinking about saying "has pain disrupted your resting time?" because some people sleep during the day, some sleep during the night, it depends. Alternatively, say when you are sleeping"—ES3

"If somebody is working throughout the night and sleeping during the day, we cannot ask them about days and nights. I usually tend to ask patients about their symptoms during sleep”—EP2

Another issue was raised on the activities (reading a newspaper, steering a car, and knitting) mentioned by the sixth question. Eleven participants stated that those activities are not very common anymore and not everybody tends to do them, including themselves.

"I do get tingling when I am biking, but I do not knit or read a newspaper. Moreover, I do not have numbness. I think other activities might be included in the list to make it more relevant to everyone"—EO2

*"I do not read a newspaper, but I read books, and I have tingling when I hold it for too long… also, I do not knit maybe we can add some more activities to this question"—*PC3 Further, when follow-up probes with two of the participants who answered no to this question during the interviews, it became evident that they answered no because they do not do any of these activities. *"no, as I do not read a newspaper, drive or knit"—PNC3.*

The last Relevancy issues mentioned by the participants was regarding question #7: do you have any neck pain? Although only two clinicians mentioned this, it seemed reasonable to the research team to discuss this issue and address it. The expert stated that: “*In my opinion, this question doesn’t really signify the differential diagnosis of CTS… the other one about little finger was creating a differential from ulnar nerve dysfunctions. As a clinician, I want to check for the double crush syndrome to see if the numbness is coming from the neck or the wrist and rule out brachial plexus injuries and cervical referral pain. Neck pain is widespread, and everyone gets neck pain, so I believe having a neck pain does not necessarily mean that it is not CTS …*”*—*EP3.

3. *Inadequate response definition*: Refers to when participants state that a question does not have enough response options for them to be able to accurately represent their signs and symptoms [16]. Overall, this theme was mentioned three times by the experts (4%), and they believed that question #4 could provide more response options to the respondents. Firstly, they recognized relieving maneuvers as a set of exercises, stretching, and wearing braces; *"also, you might get other non-movement related things that might alleviate pain as well. It might be helpful if you want to see what alleviates symptoms to CTS, to see if they are doing any non-movement related, like wearing a brace or not moving the hand at all"—*ES3. Secondly, the experts believed that the question must be asking *‘tingling OR numbness’* instead of *‘and’,* therefore providing more response options for the respondents.
4 *Perspective modifiers*: Represents when respondents of a questionnaire respond differently to one question, based on their life experience, and personal or environmental factors [16]. Three participants (4%) of the expert group, proposed that the concepts of ‘*pain’* and ‘*severe’* in questions One and Eight*,* respectively, are not well-defined, and need to be more clearly stated.

“The concept of pain is not clear, and it might confuse different people based on their perception of pain”—EP3

"The word ‘severe' is not well-defined. Some people who might be experiencing tingling and numbness during pregnancy, but it might not be severe, or they do not see it as severe, and they would answer ‘no' to this question"—EO1

"Definition of the word ‘severe' must be better stated; it might mean different things to different people"—EO2

5. *Reference point*: This theme is defined as when respondents have shifted their reference points and have difficulty calibrating their responses to a question [16]. It was the least prevalent theme that occurred in this study and only two of the participants in the non-CTS category mentioned that their reference points have changed.

“And, I do not have tingling when I drive because I do not tend to drive long distances anymore or rest my arms in driving if it is more than 30 min”—PNC4 response to question #6.

“No. I should not say no, though, because it helped at night, but it did not help me at work. Because I know I should have worn them at work, but it was too difficult, so I am not using them at work…"—PNC1 response to question #9.

### Participants’ opinion on content coverage

At the end of each interview session, the participants were asked to provide feedback on the content coverage of the Kamath and Stothard Questionnaire. All of the participants stated that this tool was brief and comprehensive, addressing most of the areas that are important for the diagnosis of CTS (sensory symptoms and pain). The main area that was not adequately addressed according to the interviews (mentioned by nine participants) was related to functional limitations (i.e., weakness of the grip strength, loss of dexterity, and dropping items) and muscle wasting that occur with chronic and severe stages of CTS [4, 6, 20].

## Discussion

According to the Consensus-based Standards for the selection of health Measurement Instruments (COSMIN), content validation must appraise both the relevancy and the comprehensiveness of a tool [21]. This CI study identified many issues that could compromise the misinterpretation and response errors to a CTS diagnostic questionnaire, which would potentially reduce the content validity and diagnostic accuracy of this tool.

Our study suggested that improvements might be made to clarity and specificity of items which would potentially improve the diagnostic performance of a revised CTS diagnostic questionnaire. The first improvement suggested was based on potentially improving existing items (Table 3). Following is a question by question analysis of the questionnaire, along with the main themes that emerged for each question, and rationale for the proposed modifications.

**Table 3:** Modified versus original questions on Kamath and Stothard questionnaire

Question #	Original questions	Modified questions
1	Has pain in the wrist woken you at night	Do you wake up because of pain in your wrist?
2	Has tingling and numbness in your hand woken you during the night?	Do you wake up because of tingling or numbness in your fingers?
3	Has tingling and numbness in your hand been more pronounced first thing in the morning	Do you have tingling or numbness in your fingers when you first wake up?
4	Do you have any trick movements to make the tingling, numbness go from your hands?	Do you have any quick movements or positions that relieve your tingling or numbness?
5	Do you have tingling and numbness in your little finger any time	Do you have numbness or tingling in your little (small/pinky) finger?
6	Has tingling and numbness presented when you were reading a newspaper, steering a car or knitting	Do certain activities (for example, holding objects or repetitive finger movement) increase the numbness or tingling in your fingers?
7	Do you have any neck pain?	Do you often have neck pain?
8	If applicable has the tingling and numbness in your hand been severe during pregnancy	Did you have numbness or tingling in your fingers when you were pregnant? (If relevant)
9	Has it helped the tingling and numbness on wearing a splint on your wrist	Have your symptoms improved with using a wrist support brace or splint? (if relevant)

### Q1: Has pain in the wrist woken you at night?

Due to the possibility of a prolonged poor posture of wrist (excessive flexion or extension during sleeping at night, the pain sensation is often considered to be nocturnal [22]; however, according to the results of the interviews of the present study, specifying the time of the sleep might cause a reduction in the generalizability and relevancy of the question. Many people tend to work at nights and asking about their sleeping at night might lead to confusion and misinterpretation of the question. The data from this study might justify the poor specificity (44%) and negative predictive values (34%) of the Edwards study; To address the issues raised on the clarity and relevancy of this question, our research team suggested modifying this question to: “*Do you wake up because of pain in your wrist?”*

### Q2: Has tingling and numbness in your hand woken you during the night?

The feeling of pins and needles, with or without numbness and sleep disturbances are identified as the most common feature of those presenting with CTS [4, 6, 23]. The theories regarding sleep disturbance are controversial, but one of the widely accepted ones is fluid retention or redistribution of body fluids while sleeping [24] (or generally in the lying position). Lying posture together with the wrist flexion or extension increase the pressure in the carpal tunnel and on the median nerve, therefore exacerbating the tingling and numbness associated with CTS [24]. All of the participants of this study found question two as the most critical question to be asked for the diagnosis of CTS. To address the relevancy issues raised with asking about the *night,* we proposed modifying the question to: *“*Do you wake up because of tingling or numbness in your fingers?”

### Q3: Has tingling and numbness in your hand been more pronounced first thing in the morning.

The theory behind the increase of non-painful disturbances and pins and needles sensations during sleep time has already been discussed in explanations of the first and second questions. This question has been proved to have moderate to strong positive and negative predictive values [25]. The word *presented* caused comprehension issues and made this question more difficult to understand. Also asking about *morning* makes this question less relevant to the large target group of people with CTS*.* To address these issues, the research team have proposed modifying this question to:” Do you have tingling or numbness in your fingers when you first wake up?”

### Q4: Do you have any trick movements to make the tingling, numbness go from your hands?

The rationale behind including this question is another feature of CTS, relating to the alleviation of the symptoms of CTS with shaking, resting their hands when driving or performing manual activities, and hanging out of the side of the bed [20]. All of the participants CTS group of this study (n = 5) stated that they have developed a form of relieving technique, which included mild exercises, stretching, shaking or immobility; whether it abated the symptoms immediately or not. The current iteration of this question implies that it is solely asking about an immediate improvement of symptoms by minimum activity and has a low negative predictive value (42%) [25]. This question could be more comprehensive to include other movements, and non-movement positions that relieve the symptoms of CTS; therefore, we modified it to: *“Do you have any quick movements or positions that relieve your tingling or numbness?”*

### Q5: Do you have tingling and numbness in your little finger any time?

This question discusses the sensory distribution of the ulnar nerve, which is one of the main differentials of CTS [6, 24]. Classic CTS usually involves sensory deficits of the median nerve which are the first three and a half fingers (not the fifth digit). The clarity issue raised on this item was addressed by just adding a short explanation of the anatomical location of the little finger: *“Do you have numbness or tingling in your little (small/pinky) finger?”*

### Q6: Has tingling and numbness presented when you were reading a newspaper, steering a car or knitting?

This question is developed based on the exacerbation of the CTS signs and symptoms by holding the wrist in flexion or extension position [22]. The pressure within the carpal tunnel has been proved to increase with a deviation of the wrist from the neutral position and is directly associated with CTS [26]. This item is the only question of the KSQ which is measuring the functional aspects associated with CTS. All of the activities mentioned in this question include sustained wrist flexion or extension; however, based on the finding of this study, they might not be necessarily relevant to all of the respondents. Therefore, we decided to modify it to: “*Do certain activities (for example, holding objects or repetitive finger movement) increase the numbness or tingling in your fingers?”*

### Q7: Do you have any neck pain?

This question is aiming to exclude several differential diagnoses of CTS, including cervical neuropathy and brachialgia. Pressure at any point on the brachial plexus might lead to a sensation of tingling and numbness on the median nerve innervated area; therefore, it is mandatory to rule out other diagnoses which have similar manifestations as CTS. The question was formatted as: *“do you often have neck pain?”* to better correlate the occurrence of neck disorders with CTS.

### Q8: If applicable has the tingling and numbness in your hand been severe during pregnancy?

Pregnancy is one of the most well-known risk factors for CTS. The prevalence of CTS among pregnant women has been reported to be 34% in a cohort of 639 participants [27]. Increased blood pressure during pregnancy due to hormonal changes seems to increase the pressure in the carpal canal, leading to subsequent feelings of CTS signs and symptoms [27]. This question was not relatable to any of the participants of the current study. Further analysis may calculate psychometric properties of the KSQ, incorporating two different versions, with and without this question. Meanwhile, to tackle the RP issues raised about the concept of *severe* in this study, it was reworded to: *“Did you have numbness or tingling in your fingers when you were pregnant? (If relevant)”.*

### Q9: Has it helped the tingling and numbness on wearing a splint on your wrist?

Immobilization of the wrist and maintaining it in a neutral position decreases the pressure in the carpal canal and is the basis of using a splint [26]. Furthermore, systematic reviews have indicated that night splinting (orthoses) with the wrist in neutral position is effective [28]. According to the findings of the present study, the word *splint* causes comprehension issues; therefore, we modified it to: *“Have your symptoms improved with using a wrist support brace or splint? (if relevant)”.*

### Recommendations on potential addition of questions to the Kamath and Stothard questionnaire

Kamath and Stothard questionnaire seem to be missing one important factor of the Levine’s questionnaire. A simple look at the KSQ, it is evident that most of the questions are biased towards examining sensory symptoms, i.e. pain, numbness, tingling [8]. Despite the frequent complaints of the weakness and functional limitations of the CTS patients, only one question (question 6) addresses this construct.

Given the KSQ questions with the most confusion in our cognitive interviews are those with the poorest diagnostic accuracy in previous studies [25], changes to this questionnaire are needed. Further important items were identified from the interviews that may have diagnostic value. Although cognitive interviewing can guide proposed changes, future investigations on reliability, validity and diagnostic accuracy would be needed before any recommendations for use could be made. In total we suggested three new potential questions which are listed below and are presented in Table 4. The usefulness of the incorporation of these recommended questions as well as the best order for them is pending further analysis.

1. *Is your numbness or tingling mainly in your thumb, index, and/or middle finger?* We recommend the addition of this question for a more specific approach to the measurement of the median nerve distribution area. It could also be substituted with the second question, asking about tingling and numbness sensation in hands.
2. *Do you drop small objects like coins or keys?* There is only one question asking about function on the KSQ, and as mentioned earlier, the function is one of the main constructs of the Levine’s questionnaire [29]. The participants of our study recommended the incorporation of more questions on functional limitations and impairments.
3. *Do you have numbness or tingling in your toes?* One of the main differential diagnoses for CTS is peripheral neuropathy, i.e. diabetes. The prevalence of CTS in those having diabetic neuropathies is reported to be high (30%), with the pathophysiologic mechanisms being very complex and not yet fully understood [6]. Adding a comparative body location might improve the specificity of this questionnaire.

**Table 4:** Suggested modified questionnaire

1	Do you wake up because of pain in your wrist?	Yes	No
2	Do you wake up because of tingling or numbness in your fingers?	Yes	No
3	Do you have tingling or numbness in your fingers when you first wake up?	Yes	No
4	Is your numbness or tingling mainly in your thumb, index, and/or middle finger?	Yes	No
5	Do you have any quick movements or positions that relieve your tingling or numbness?	Yes	No
6	Do you have numbness or tingling in your little (small/pinky) finger?	Yes	No
7	Do certain activities (for example, holding objects or repetitive finger movement) increase the numbness or tingling in your fingers?	Yes	No
8	Do you drop small objects like coins or a cup?	Yes	No
9	Do you often have neck pain?	Yes	No
10	Did you have numbness or tingling in your fingers when you were pregnant? (If relevant)	Yes	No
		Not relevant to me	
11	Do you have numbness or tingling in your toes?	Yes	No
12	Have your symptoms improved with using wrist support brace or splint? (If relevant)	Yes	No
		Not relevant to me	

### Recommendation on complementing the Kamath and Stothard Questionnaire with Katz and Stirrat hand symptoms diagram

**Fig. 1:**
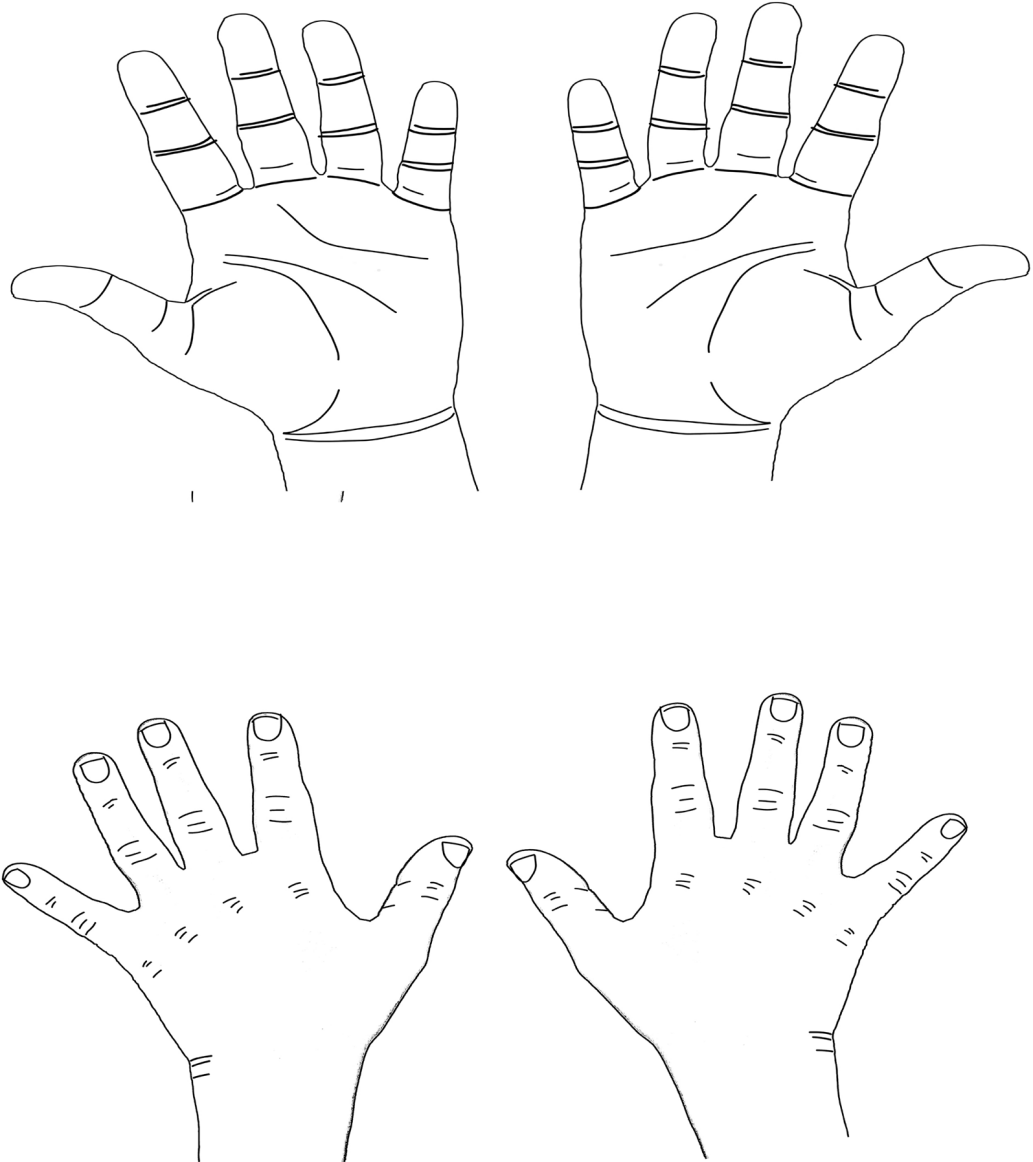
Katz and Stirrat hand symptoms diagram

Incorporation of a hand symptoms diagram with the KSQ is suggested to complement the screening questions with a visual map of the symptoms since they provide complementary information. Katz and Stirrat hand diagram (Fig. 1) is proved to have high diagnostic accuracy when combined with other clinical examination tests [30]. The following is an instruction that make for a better flow of the questionnaire, followed by the modified version of the questionnaire (Table 4) and a hand symptoms diagram (Fig. 1). One must be aware that this combination of hand diagram and questionnaire are pending future studies to establish psychometric properties and diagnostic accuracy.

#### Instructions

Please answer the following questions as yes or no. We will ask about numbness which some people describe as having no feeling or dead feeling. We will also ask about tingling which some people call pins and needles or prickly feelings. Please pick the answer about how your hand has felt over the last month. Also, by shading the diagram below, please show where you have experienced numbness, tingling, or pain.

### Limitations and future research

One interviewer did all of the interviews, consequently transcribed the interviews, and did the analysis. This might have potentially introduced bias. To justify this shortcoming, the findings were discussed iteratively with a coauthor (JM) who reviewed and confirmed all of the coding for the content analysis. Further, all of the suggested modifications of the KSQ were discussed and established in two group meetings with at least two graduate students and two of the research team members. Another limitation of the current study is that all of the participants had to speak English to be eligible to participate. This in turn, would limit the generalizability of the results of this study. Lastly, we did not include participants with cognitive impairments. Future research should assess the comprehensibility of the KSQ in a sample of cognitively impaired persons, experiencing symptoms of CTS.

Future research should assess the diagnostic accuracy of the modified KSQ. The modified KSQ should not be used by the clinicians until the psychometric properties are established. Testing the additional value of the incorporation of the Katz hand symptoms diagram with the KSQ in the clinics is also pending future research.

## Conclusion

This study found multiple areas of uncertainty that could contribute to measurement error on a questionnaire designed for the diagnosis of CTS. Several errors occurred in the interviews, and the content validity of the current iteration of the Kamath and Stothard was not established. Most of the errors were regarding the Comprehension, Clarity, and Relevancy of the questions. Cognitive interviewing guided options for potential improvements in the wording of the KSQ, which should be tested in future studies. The findings of this study could be potentially useful for CTS diagnosis and assist the clinicians working in the field of hand, such as hand therapists, hand surgeons, orthopedic surgeons, and family physician.

## Data Availability

All data generated or analyzed during this study are included in this published article.

## Appendix 1: KAMATH and STOTHARD Questionnaire

- HISTORY (circle Yes/No number).
- Has pain in the wrist woken you at night.
- Yes 1 No 0.
- Has tingling and numbness in your hand woken you during the night.
- Yes 1 No 0.
- Has tingling and numbness in your hand been more pronounced first thing in the morning.
- Yes 1 No 0.
- Do you have any trick movements to make the tingling, numbness go from your hands.
- Yes 1 No 0.
- Do you have tingling and numbness in your little finger any time.
- Yes 0 No 3.
- Has tingling and numbness presented when you were reading a newspaper, steering a car or knitting.
- Yes 1 No 0.

Do you have any neck pain.

- Yes _1 No 0.

If applicable has the tingling and numbness in your hand been severe during pregnancy.

- Yes 1 No _1 N/A 0.

Has it helped the tingling and numbness on wearing a splint on your wrist.

- Yes 2 No 0 N/A 0.

Total.

**Reference**

1. Kamath V, Stothard J. A clinical questionnaire for the diagnosis of carpal tunnel syndrome. *J Hand Surg Am*. 2003;28 B(5):455–459. 10.1016/S0266-7681(03)00151-7

## Appendix 2: Pre-planned probes

Practice question:

What job do you think you’d be terrible at?

Probes:

- How do you define “job”?
- How do you define “terrible”?

Question 1. Has pain in the wrist woken you at night.

- Say it in your own words please, or what do you think that item means, or what does that mean to you, or can you repeat the question I just asked in your own words?
- How did you arrive at that answer? Or, why did you say yes, no?
- Was that easy or hard to answer?
- I noticed that you hesitated—tell me what you were thinking
- What does the term “wrist” mean to you?
- What does the term “pain” mean to you? How do you describe (define) “pain”?
- What’s at night? How do you define night?
- Is there anything else besides pain that you think has an impact on your sleep?
- How do you remember that the pain has woken you at night?

Question 2. Has tingling and numbness in your hand woken you during the night.

- Say it in your own words please, or what do you think that item means, or what does that mean to you, or can you repeat the question I just asked in your own words?
- How did you arrive at that answer?
- Was that easy or hard to answer?
- I noticed that you hesitated—tell me what you were thinking
- What does the term “tingling” mean to you?
- What does the term “numbness” mean to you?

Question 3. Has tingling and numbness in your hand been more pronounced first thing in the morning.

- Say it in your own words please, or what do you think that item means, or what does that mean to you, or can you repeat the question I just asked in your own words?
- How did you arrive at that answer?
- Was that easy or hard to answer?
- I noticed that you hesitated—tell me what you were thinking
- What does the term “hand” mean to you? How do you describe (define) “hand”?
- What does the term “morning” mean to you? How do you describe (define) “morning”?

Question 4. Do you have any trick movements to make the tingling, numbness go from your hands.

- Say it in your own words please, or what do you think that item means, or what does that mean to you, or can you repeat the question I just asked in your own words?
- How did you arrive at that answer?
- Was that easy or hard to answer?
- I noticed that you hesitated—tell me what you were thinking

Question 5. Do you have tingling and numbness in your little finger any time.

- Say it in your own words please, or what do you think that item means, or what does that mean to you, or can you repeat the question I just asked in your own words?
- How did you arrive at that answer?
- Was that easy or hard to answer?
- I noticed that you hesitated—tell me what you were thinking
- What does the term “little finger” mean to you? How do you describe (define) “little finger”?

Question 6. Has tingling and numbness presented when you were reading a newspaper, steering a car or knitting.

- Say it in your own words please, or what do you think that item means, or what does that mean to you, or can you repeat the question I just asked in your own words?
- How did you arrive at that answer?
- Was that easy or hard to answer?
- I noticed that you hesitated—tell me what you were thinking

Question 7. Do you have any neck pain.

- Say it in your own words please, or what do you think that item means, or what does that mean to you, or can you repeat the question I just asked in your own words?
- How did you arrive at that answer?
- Was that easy or hard to answer?
- I noticed that you hesitated—tell me what you were thinking
- What does the term “neck” mean to you? How do you describe (define) “neck”?

Question 8. If applicable has the tingling and numbness in your hand been severe during pregnancy.

- Say it in your own words please, or what do you think that item means, or what does that mean to you, or can you repeat the question I just asked in your own words?
- How did you arrive at that answer?
- Was that easy or hard to answer?
- I noticed that you hesitated—tell me what you were thinking
- What does the term “severe” mean to you? How do you describe (define) “severe”?

Question 9. Has it helped the tingling and numbness on wearing a splint on your wrist.

- Say it in your own words please, or what do you think that item means, or what does that mean to you, or can you repeat the question I just asked in your own words?
- How did you arrive at that answer?
- Was that easy or hard to answer?
- I noticed that you hesitated—tell me what you were thinking
- What does the term “wrist” mean to you? How do you describe (define) “wrist”?

Additional questions:

Do you feel you had enough questions to describe your experience? Were there too many questions? Not enough?

Is there anything about your symptoms that you feel was not covered by these questions today?

Did you find any of the questions difficult to understand?

**References**

1. Willis GB. Cognitive Interviewing. A “how to” guide. *Evaluation*. 1999:1–37. 10.1525/jer.2006.1.1.9.
2. Packham, T., MacDermid, J., Michlovitz, S. and Buckley, N. (2018). Content validation of the Patient-Reported Hamilton Inventory for Complex Regional Pain Syndrome. *Canadian Journal of Occupational Therapy*, 85(2), pp.99–105.

## Abbreviations

CTS: Carpal tunnel syndrome
KSQ: Kamath and Stothard questionnaire
CI: Cognitive interview
PT: Physical therapist
OT: Occupational therapist
RP: Reference point
PM: Perspective modifier
IRD: Inadequate response definition
SD: Standard deviation
EO: Expert OT
EP: Expert PT
ES: Expert student
PC: Patients with CTS
PNC: Patients without CTS
